# Corticospinal MRI tractography in space-occupying brain lesions by diffusion tensor and kurtosis imaging methods

**DOI:** 10.1186/2197-7364-2-S1-A82

**Published:** 2015-05-18

**Authors:** Joao Leote, Rita Nunes, Luis Cerqueira, Hugo Alexandre Ferreira

**Affiliations:** epartment of Neurosurgery, Hospital Garcia de Orta, Almada, Portugal; Institute of Biophysics and Biomedical Engineering, Faculty of Sciences of the University of Lisbon, Lisboa, Portugal

Recently, DKI-based tractography has been developed, showing improved crossing-fiber resolution in comparison to deterministic DTI-based tractography in healthy subjects. In this work, DTI and DKI-based tractography methods were compared regarding the assessment of the corticospinal tract in patients presenting space-occupying brain lesions near cortical motor areas. Nine patients (4 males) aged 23 to 62 years old, with space-occupying brain lesions (e.g. tumors) were studied for pre-surgical planning using a 1.5T MRI scanner and a 12-channel head coil. In 5 patients diffusion data was acquired along 64 directions and in 4 patients along 32 directions both with b-values 0, 1000 and 2000 s/mm2. Corticospinal tracts were estimated using deterministic DTI and DKI methods and also using probabilistic DTI. The superior cerebellar peduncles and the motor cortical areas, ipsilateral and contralateral to the lesions, were used as seed regions-of-interest for fiber tracking. Tracts courses and volumes were documented and compared between methods. Results showed that it was possible to estimate fiber tracts using deterministic DTI and DKI methods in 8/9 patients, and using the probabilistic DTI method in all patients. Overall, it was observed that DKI-based tractography showed more voluminous fiber tracts than when using deterministic DTI. The DKI method also showed curvilinear fibers mainly above lesions margins, which were not visible with deterministic DTI in 5 patients. Similar tracts were observed when using probabilistic DTI in 3 of those patients. Results suggest that the DKI method contribute with additional information about the corticospinal tract course in comparison with the DTI method, especially with subcortical lesions and near lesions’ margins. Therefore, this study suggests that DKI-based tractography could be useful in MRI and hybrid PET-MRI pre-surgical planning protocols for improved corticospinal tract evaluation.

